# Global hotspots and trends in microbial-mediated grassland carbon cycling: a bibliometric analysis

**DOI:** 10.3389/fmicb.2024.1377338

**Published:** 2024-04-29

**Authors:** Xing Xiang, Tuo Yao, Baiying Man, Dong Lin, Changning Li

**Affiliations:** ^1^College of Grassland Science, Gansu Agricultural University, Lanzhou, China; ^2^College of Life Science, Shangrao Normal University, Shangrao, China; ^3^Key Laboratory for Regional Plants Conservation and Ecological Restoration of Northeast Jiangxi, College of Life Science, Shangrao Normal University, Shangrao, China; ^4^Key Laboratory of Grassland Ecosystem, Gansu Agricultural University, Ministry of Education, Lanzhou, China

**Keywords:** bibliometric analysis, grassland, carbon cycling, microorganism, R software

## Abstract

Grasslands are among the most widespread environments on Earth, yet we still have poor knowledge of their microbial-mediated carbon cycling in the context of human activity and climate change. We conducted a systematic bibliometric analysis of 1,660 literature focusing on microbial-mediated grassland carbon cycling in the Scopus database from 1990 to 2022. We observed a steep increase in the number of multidisciplinary and interdisciplinary studies since the 2000s, with focus areas on the top 10 subject categories, especially in Agricultural and Biological Sciences. Additionally, the USA, Australia, Germany, the United Kingdom, China, and Austria exhibited high levels of productivity. We revealed that the eight papers have been pivotal in shaping future research in this field, and the main research topics concentrate on microbial respiration, interaction relationships, microbial biomass carbon, methane oxidation, and high-throughput sequencing. We further highlight that the new research hotspots in microbial-mediated grassland carbon cycling are mainly focused on the keywords “carbon use efficiency,” “enzyme activity,” “microbial community,” and “high throughput sequencing.” Our bibliometric analysis in the past three decades has provided insights into a multidisciplinary and evolving field of microbial-mediated grassland carbon cycling, not merely summarizing the literature but also critically identifying research hotspots and trends, the intellectual base, and interconnections within the existing body of collective knowledge and signposting the path for future research directions.

## 1 Introduction

As one of the five major biomes worldwide, grassland is open regions that are predominately occupied by herbaceous plants and characterized by a dry climate. There are three types of grassland, including tropical zonal grassland (near the equator and are interspersed with scatted trees, such as the middle African savannas, the Oceania mallee, and the South American campo), temperature zonal grassland (farther from the equator and receive less precipitation than tropical zonal grassland, such as the European steppes and the North American prairies), and alpine and polar grassland (in high elevation and high latitude areas, such as alpine meadows and tundra in the Tibet plateau) (Zhao et al., 2020; Dong, 2022). The various types of grassland cover an area of 52.5 million km^2^, accounting for 40.5% of ice-free terrestrial land (Zhao et al., 2020), and guarantee the essential ecological function of terrestrial carbon reservoirs by storing ~40% of global soil carbon stock above (biomass) and below ground (biomass and soil organic carbon), with the latter contributing to more than 90% of the total amount (Bai and Cotrufo, 2022). In addition, grassland has been regarded as an important biotic sink of CH_4_ due to its ability to uptake a large amount of atmospheric CH_4_ (Bender and Conrad, 1992; Wang et al., 2014; Wei et al., 2015; Yu et al., 2017; Xu et al., 2022).

Grassland microorganisms play a key role in mediating the grassland carbon cycle. After photosynthesis, the majority of photosynthate is translocated belowground to be respirated by plant roots and microorganisms dwelling in narrow areas in the vicinity of the root, such as mycorrhizal fungi, which establish mutational symbiosis with various host plants and receive organic compounds from the photosynthetic host in exchange for mineral nutrients (Genre et al., 2020). This CO_2_ release based on newly assimilated carbohydrates is referred to as autotrophic respiration. Correspondingly, heterotrophic respiration integrates the respiration of free-living microorganisms primarily involved in litter degradation (older carbon) in the soil, such as saprotrophic fungi and Gram-positive bacteria (Brüggemann et al., 2011; Yu and Dijkstra, 2020). The total CO_2_ flux of autotrophic and heterotrophic respirations is one of the largest fluxes between the atmosphere and terrestrial ecosystems (Raich et al., 2002). Many studies have found the two components of respiration have distinct response to changing environmental variables; for example, the former is closely coupled with the supply and allocation of assimilate, while the latter is mainly affected by temperature, soil moisture, and substrate availability (Allison et al., 2010; Brüggemann et al., 2011; Nie et al., 2013). Microorganisms not only accelerate carbon cycling by respiration but are also associated with carbon stabilizing and sequestration by catabolic and anabolic metabolism. On one hand, they produce extracellular enzymes to attract and alter plant litter chemically, resulting in the formation of refractory residues that are not readily utilized by microorganisms and deposited in the soil. On the other hand, these easily degraded and accessible molecules are converted into microbial necromass via uptake-biosynthesis-death-lysis, which contributes to microbial-derived carbon deposition in soil (Liang et al., 2017, 2019). The fungal and bacterial necromass have been considered to be major C-containing components contributing to long-lasting soil organic matter, while the ratio of fungi-to-bacteria necromass carbon ranges from 1.2 to 4.1 across global grassland, suggesting fungi contribute more significantly to grassland soil carbon sequestration compared to those of bacteria (Bai and Cotrufo, 2022).

Although the field of microbial-mediated grassland carbon cycling has received significant attention and is involved in multidisciplinary subjects, there have been few studies focusing on the following aspects: (1) What is the current intellectual landscape of microbial-mediated grassland carbon cycling research? (2) Which subject categories are involved in this field? (3) Which key players—authors, institutions, and countries—are driving the development of microbial-mediated grassland carbon cycling research? (4) Which journals and publications have the highest academic contribution and research impact? (5) What are the hotspots and critical turns that underpin the advancements in the field of microbial-mediated grassland carbon cycling research? To approach these questions, we undertook a systematic bibliometric analysis over the past decades across a breadth of publications focused on the field of microbial-mediated grassland carbon cycling. By applying bibliometric and visual analysis techniques, it provides a comprehensive overview of the intellectual landscape from a global perspective for the study of microbial-mediated grassland carbon cycling. Our findings offer a theoretical foundation and valuable scientific insights for further research on these topics, while also highlighting the research hotspots and emerging development trends that warrant deeper investigation.

## 2 Materials and methodology

### 2.1 Data collection

Scopus (https://www.elsevier.com/solutions/scopus), created by Elsevier in November 2004, is currently the largest bibliographic database available on the information market. This database currently indexes more than 87 million documents from over 25,000 active titles and 7,000 publishers worldwide. The Scopus database provides a broad perspective on research output across various disciplines related to health, life, social, and physical sciences. To ensure that only relevant results are included, we employed the Scopus database and implemented a key field retrieval strategy as our selection method. The retrieval condition is KEY = ((grassland OR rangeland OR steppe OR savanna OR prairie OR mallee OR campo OR pampas OR tundra OR meadow) AND (microb^*^ OR microo^*^ OR fung^*^ OR ^*^archae^*^ OR ^*^bacter^*^ OR virus) AND (carbon OR metha^*^ OR CH_4_ OR CO_2_)), with time span of 1990–2022 and document types limited to article and review. The specific condition is set to acquire relevant results on microbial-mediated grassland carbon cycling. The obtained literatures, along with all information, were downloaded and saved in the form of BibTeX for subsequent bibliometric analysis.

### 2.2 Methodology

#### 2.2.1 Bibliometric tools and visualization

Bibliometrix package of R software is one of the most popular tools utilized for literature bibliometric analysis (Aria and Cuccurullo, 2017). It provides a comprehensive range of functionalities for analyzing bibliometric data, including data loading and conversion, creating matrix of documents × attributes, generating network matrices, performing co-occurrence and collaboration network analysis, co-citation analysis, and clustering analysis. This tool offers researchers a convenient and assessable means of conducting bibliometric analysis. In this study, the Bibliometrix (version: 4.0.1) package was employed to analyze the research situation and development trends in the field of microbial-mediated grassland carbon cycling, especially focusing on evaluating literature quality, research power, identifying research hotspot, and tracking theme evolution. The descriptive results of bibliometric analysis were visualized using the ggplot2 (version: 3.4.2) package (Wickham, 2011), and the collaboration and co-occurrence results of the bibliometric analysis were visualized using Cytoscape software (version: 3.9.1) (Shannon et al., 2003). The burst detection of keywords was analyzed by Sci2 software (version: 1.3.0) (Sci2 Team, 2009) with a gamma value of 1.0 and subsequently visualized by the ggplot2 package. The word clouds of keywords were created by the website of WordArt (https://wordart.com/) with the Tex Gyre Bonum font. The statistical R package changepoint.np (version: 1.0.5) (Haynes et al., 2016) was used to access the change in the annual number of documents and divide the research time zone.

#### 2.2.2 Technology classification by document type

This study employed the four-digit ASJC code to categorize subject, enabling Scopus to classify the scientific field of published research. ASJC codes were assigned to each journal, and each document was categorized based on the corresponding ASJC code of the journal in which it was published. The ASJC consists of a three-level classification system, including four supergroups (health sciences, life sciences, social sciences, and physical sciences), 27 subject areas, and 334 fields. Physical sciences encompass 10 subject areas, social sciences encompass six, and life sciences and health sciences each encompass five subject areas. In addition, there is multidisciplinary subject area that does not fall under any of the four supergroups.

#### 2.2.3 Activity index and attractive index

The activity index (AI) and attractivity index (AAI) were used to evaluate the relative effort and importance of a given country accordingly, building upon the previous studies (Chen and Guan, 2011; Huang et al., 2020). The two indexes were calculated based on the following formulas:

The activity index (AI):


(1) $$\begin{array}{l} AI_{i}^{t}=\frac{P_{i}^{t}/\sum P}{TP^{t}/\sum TP} \end{array}$$

The attractive index (AAI):


(2) $$\begin{array}{l} AAI_{i}^{t}=\frac{C_{i}^{t}/\sum C}{TC^{t}/\sum TC} \end{array}$$

Within the two Equations (1, 2), $P_{i}^{t}\;and\;C_{i}^{t}$ represent the publication volume and citation number for country *i* in year *t*, ∑*P* and ∑*C* stand for the total number of publications and citations for country *i* over a specified time period. Additionally, *TP*^*i*^ and *TC*^*i*^ signify the global sum of publications and citations in year *t*, ∑*TP* and ∑*TC* represent the global total number of publications and citations during the same time period with ∑*P* and ∑*C*, respectively. To visually represent the resulting AI and AAI values, quadrant diagram was used. In this diagram, the reference lines were placed at $AI_{i}^{t}=1$ and $AAI_{i}^{t}=1$ to indicate the global average. Countries with $AI_{i}^{t}=1$ and $AAI_{i}^{t}=1$ would fall on the reference line, indicating their research effort and academic importance are equal to the global average. Furthermore, the balance line represented by a straight line with a slope of 1 and passing through the origin, is plotted on the diagram, symbolizing a balanced performance where the research effort ($AI_{i}^{t}$) is proportional to the academic importance ($AAI_{i}^{t}$).

## 3 Results

### 3.1 Basic characteristics of the documents

**Figure 1:**
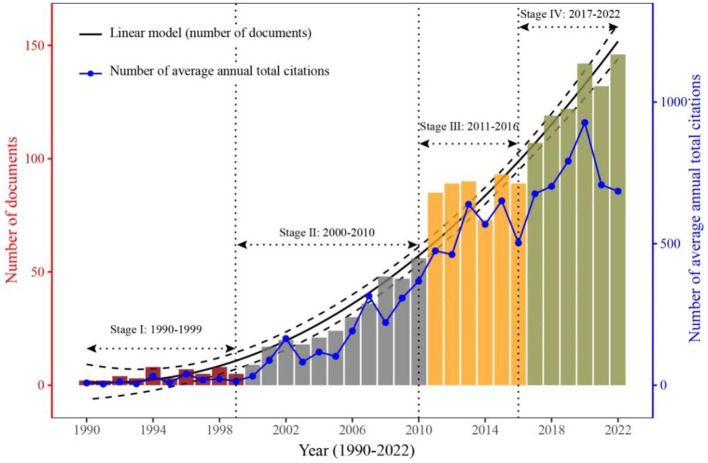
Trends in the number of documents and citations related to microbial-mediated grassland carbon cycling (Scopus, from 1990 to 2022). The number of documents (bar plot) is divided into four stages (from I to IV) with different colors. Average annual citations (line plot with blue color) are calculated by dividing the total citation by the number of years since publication.

Out of the 1,660 (article: 1,637, review: 23) studies extracted from this database, a small portion consisted of single-authored documents (34 documents, 2.05%) and dual-authored documents (160 documents, 9.64%). In contrast, the majority of the studies were multi-authored documents (1,466 documents, 88.31%). The variation trend of annual publications showed a linear growth pattern during 1990–2022, with annual growth rate of 14.35% (Figure 1). Four study stages were divided according to the turning points of publication volume (Figure 1). During Stage I (1990–1999), which was characterized as the basic research stage, the annual production remained below eight articles. However, in Stage II (2000–2010), the annual production ranged from 9 to 56 articles, indicating a significant increase in research output during that period. Stages III (2011–2016) and IV (2017–2022) show a rapid growth trend with the average annual production of 87 and 128 documents and average annual growth rate of 0.92% and 6.41%, respectively. The consistent increase in research production indicates that the field of microbial-mediated grassland carbon cycling is currently in a “growth phase” and holds substantial potential for further development.

The global citation score (GCS) of those documents was 83,586, with an average of 50.35 citations per article over the given period (1990–2022). The variation trend of average annual total citations showed a slow growth during stage I (1990–1999), with an average of 16.20 citations (Figure 1). Subsequently, in stages II and III, the research output experienced a rapid and steady increase, culminating in the highest average annual total citations recorded in 2020 (927.33 citations; Figure 1). However, there was a rapid decrease in average annual total citations after 2020, possibly due to time lag in the development of academic field and subsequent publication of literature. Nonetheless, the fourth stage had the largest mean average annual total citation count (748 citations), indicating the increasing attention focused on microbial-mediated grassland carbon cycling during this period.

#### 3.1.1 Subject categories analysis

A total of 1,660 documents have been classified into four supergroups and 23 distinct subject categories based on the ASJC classification system, i.e., life sciences (1,166, 70.24%); physical sciences (854, 51.45%); health sciences (182, 10.96%); and social sciences (66, 3.98%). Within the field of life sciences, the primary subject categories were Agricultural and Biological Sciences (1,032, 62.17%), followed by Immunology and Microbiology (410, 24.70%), and Biochemistry, Genetics, and Molecular Biology (143, 8.61%). In the discipline of Physical Sciences, the top subject categories were Environmental Science (701, 42.23%), Earth and Planetary Sciences (228, 13.73%), Chemistry (32, 1.93%), and Energy (30, 1.81%). Furthermore, 180 documents (10.84%) were affiliated with medicine, which was the primary subject category within health sciences.

**Figure 2:**
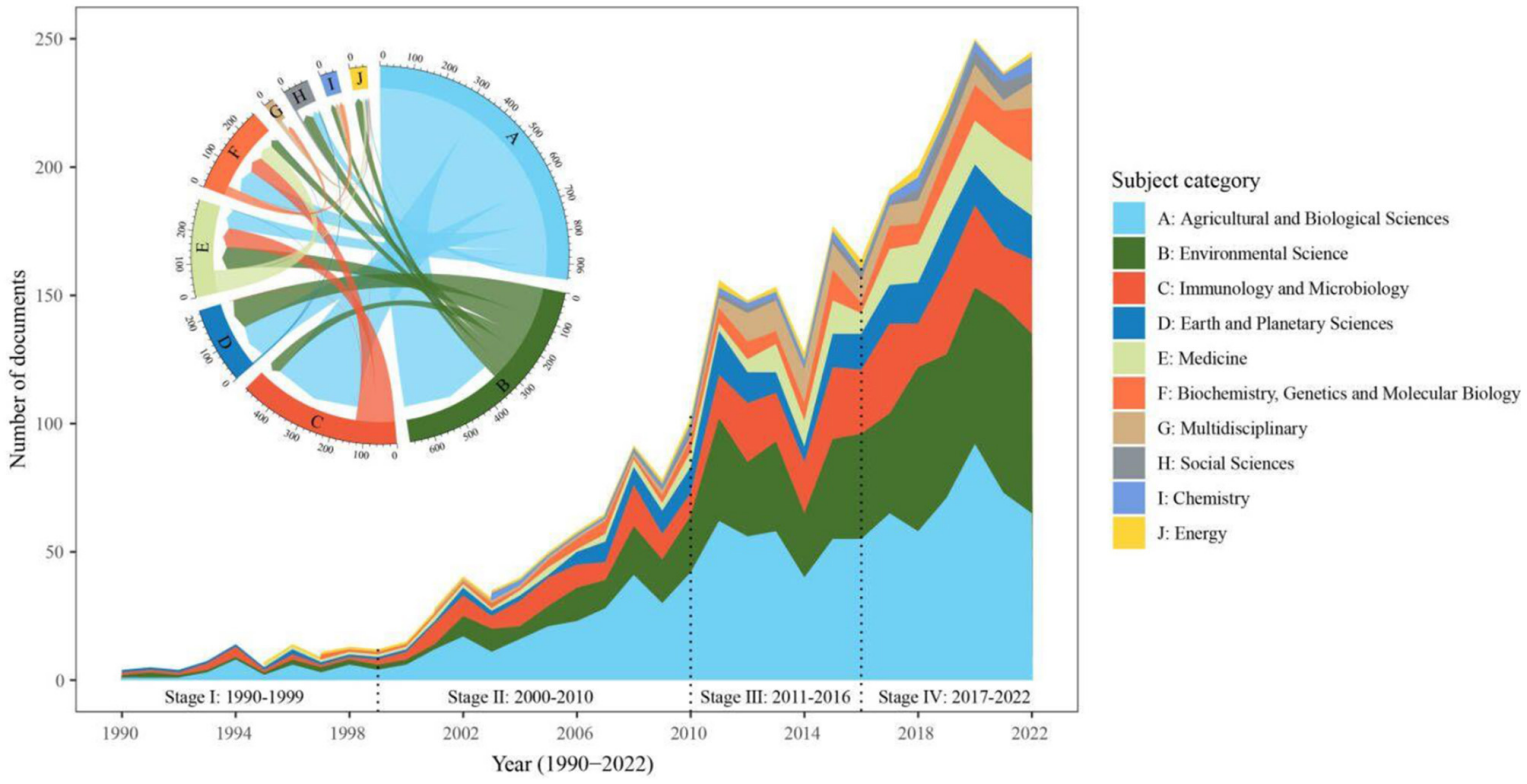
Top 10 subjects and their co-occurrence relationship in the field of microbial-mediated grassland carbon cycling (Scopus, from 1990 to 2022). Stages I–IV are same as Figure 1 and each color corresponds to a specific subject area.

The production of each subject reflects the research trend of microbial-mediated grassland carbon cycling across different domains. The publications of Agricultural and Biological Sciences presented a rapid increase during the given period (1990–2022), while those of Energy remained virtually unchanged. In terms of publication trend, the publications of Environmental Science, Immunology and Microbiology, and Earth and Planetary Sciences increased from II to III stages, whereas Biochemistry, Genetics, and Molecular Biology and Medicine increased from III to IV stages (Figure 2).

We found that the research of microbial-mediated grassland carbon cycling is a multidisciplinary and interdisciplinary field encompassing 23 different subjects, with a particular emphasis on the top 10 subject categories. The analysis of co-occurrence relationships among the top 10 subject categories revealed a total of 30 links between these subjects (Figure 2). Agricultural and Biological Sciences were closely connected with seven interconnected subjects. Environmental Science shared a strong link with Earth and Planetary Sciences (14.84%) and Medicine (11.55%; Figure 2). In contrast, Biochemistry, Genetics, and Molecular Biology exhibited strong connection with almost interconnected subjects, i.e., Agricultural and Biological Sciences (58.04%), Immunology and Microbiology (41.26%), Medicine (40.56%), Environmental Science (21.68%), Chemistry (10.49%), and Multidisciplinary (10.49%). The co-occurrence rates of Environmental Science, Immunology and Microbiology, and Earth and Planetary Sciences accounted for 32.27%, 30.52%, and 11.43%, respectively. This result indicates that effective research in the field of microbial-mediated grassland carbon cycling requires collaborative efforts across various disciplines to facilitate global investigations.

#### 3.1.2 Journal analysis

**Figure 3:**
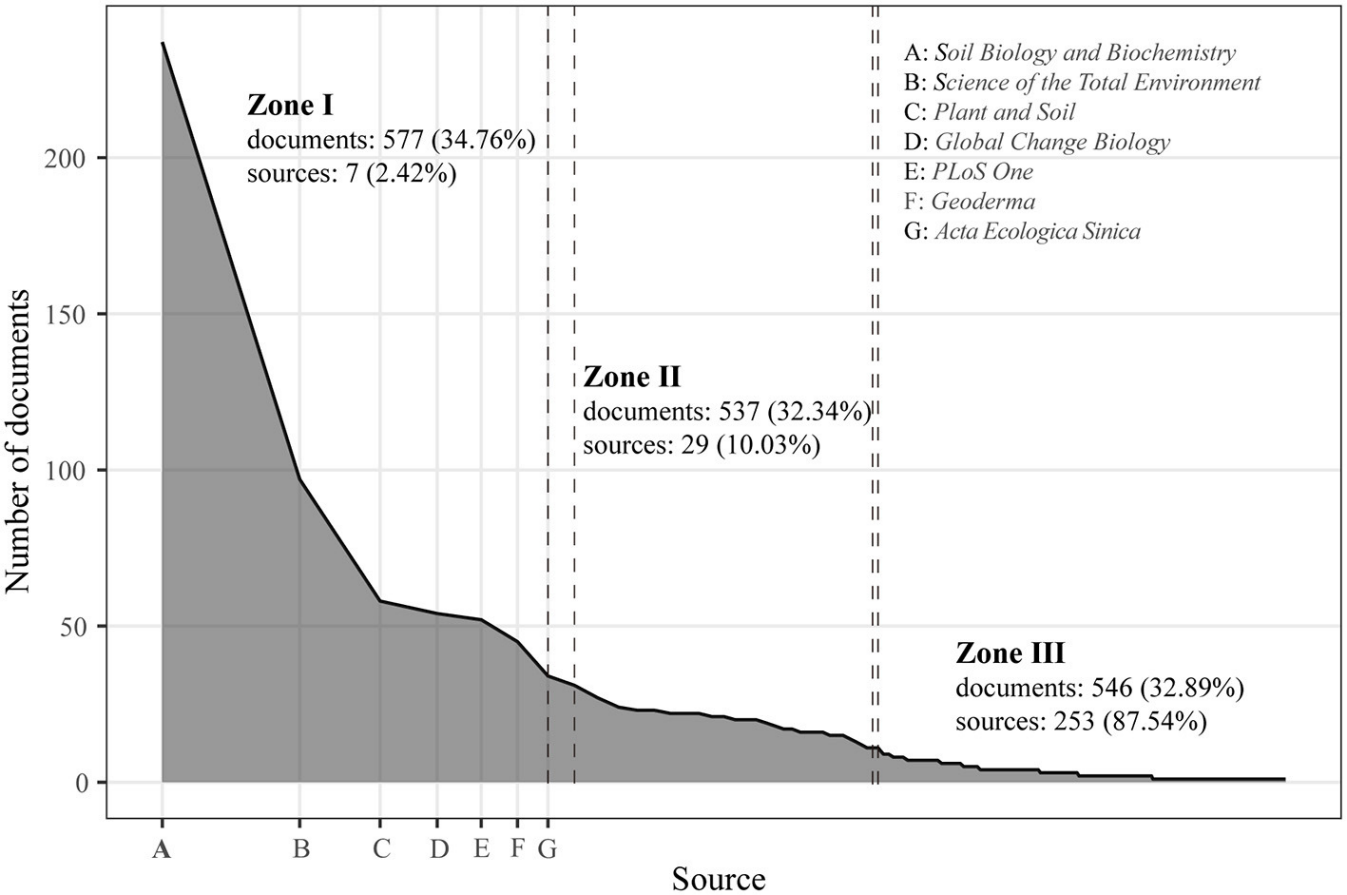
Source distribution estimation by Bradford's law for the core journal related to microbial-mediated grassland carbon cycling (Scopus, from 1990 to 2022).

A total of 1,660 documents on microbial-mediated grassland carbon cycling were published across 289 journals (Figure 3). Of them, only a small percentage (12.80%, 37 journals) carried a rather higher number of documents (>10 documents), which accumulated substantial percentage (1,125 documents, 67.77% of all documents) of total documents. In contrast, 142 journals (49.13%) only published one document. The highly disproportionate scientific production within certain fields across journals was widely observed in bibliometric analysis and is well summarized by Bradford's law.

The Bradford function from the Bibliometrix package is commonly used to identify core journals in a given field by ranking journals based on a number of published documents and trisecting them into three approximately equally populated groups, known as zones I, II, and III. Figure 3 shows that the number of journals in the three zones consisted of 7, 29, and 253 journals, respectively. Seven core journals within zone I, including *Soil Biology and Biochemistry, Science of the Total Environment, Plant and Soil, Global Change Biology, PLoS One, Geoderma*, and *Acta Ecologica Sinica*, had 577 documents and accounted for 34.76% of total documents (Figure 3). Specially, *Soil Biology and Biochemistry* published 237 documents, and *Science of the Total Environment* published 97 documents on this field. Moreover, these core journals received 30,334 global citations, which accounted for 36.29% of the total 83,586 global citations. Additionally, they received 1,373 local citations, representing 42.79% of the total 3,209 local citations (Table 1).

**Table 1:** Core journals in the field of microbial-mediated grassland carbon cycling (Scopus, from 1990 to 2022).

**Journals**	**Ps**	**GCS**	**GCS/Ps**	**LCS**	***h*-index**	**CiteScore 2022**	**SY**
*Soil Biology and Biochemistry*	237	18,393	77.6	821	71	14.3	1990
*Science of the Total Environment*	97	2,354	24.3	67	28	16.8	2008
*Plant and Soil*	58	2,207	38.1	95	27	7.8	1993
*Global Change Biology*	54	4,278	79.2	236	32	19.5	1998
*PLoS One*	52	1,678	32.3	96	26	6.0	2011
*Geoderma*	45	1,203	26.7	48	19	12.9	2006
*Acta Ecologica Sinica*	34	221	6.5	10	9	4.1	2009

Ps, number of publications; GCS, global citation scores (times); GCS/Ps, average number of citations per paper; LCS, local citation score (times); CiteScore 2022, data from the annual report of citation impact of peer-reviewed research indexed by the Scopus database; SY, start year.

Core journals identified by Bradford's function covered a wide range of disciplines, including Agricultural and Biological Sciences, Environmental Science, Immunology and Microbiology, and Multidisciplinary, which indicates the multidisciplinary nature of research on microbial-mediated grassland carbon cycling (Table 1). Furthermore, we use the average citation per document (GCS/Ps) and *h*-index to evaluate the relative importance of journals and the academic impact of documents, respectively. Compared to other journals, *Soil Biology and Biochemistry* (*h*-index: 71; GCS/Ps: 77.6) and *Global Change Biology* (*h*-index: 32; GCS/Ps: 79.2) had higher values of *h*-index and GCS/Ps, indicating that two journals have established a leading position in the field of microbial-mediated grassland carbon cycling (Table 1). This finding indicates that the topic of microbial-mediated grassland carbon cycling holds significant prominence in mainstream journals.

### 3.2 Research power of microbial-mediated grassland carbon cycling

#### 3.2.1 Country and institution analysis

##### 3.2.1.1 Quantity of articles and citations

The documents analyzed in this study were sourced from 54 countries worldwide, with the majority originating from Europe, America, Asia, and Oceania. China harbored the largest Ps (500) and the second GCS (15,381), while the USA had the largest GCS (30,408), LCS (1,243), GCS/Ps (87.9), and *h*-index (90) with the second Ps (346; Table 2). China, the USA, Spain, and the United Kingdom had the lowest values of MCP (<0.5), indicating that the majority of publications were finished within their respective countries. Other countries, including Germany, Canada, France, Australia, New Zealand, and Sweden, had the highest values of MCP (>0.5) and showed close research cooperation with other countries (Table 2). In terms of GCS/Ps measurement, the USA occupied decisive position in the ensemble of research work with the highest GCS/Ps (87.9) values, which resulted from substantial number of high-quality documents. France (69.1), the United Kingdom (66.4), and Germany (61.9) in Western Europe, Australia (58.5), and New Zealand (57.0) from Oceania emerged as countries with rather high GCS/Ps. The research history of countries seems to have a significant influence on their academic impact. In the field of microbial-mediated grassland carbon cycling, pioneer countries (the USA, France) harbored high values of GCS/Ps, while the followers (China, Sweden) had comparatively low values of GCS/Ps (Table 2).

**Table 2:** The top 10 most productive countries in the field of microbial-mediated grassland carbon cycling (Scopus, from 1990 to 2022).

**Country**	**Ps**	**MCP**	**GCS**	**LCS**	**GCS/Ps**	***h*-index**	**SY**
China	500	0.41	15,381	771	30.8	65	2005
USA	346	0.26	30,408	1,243	87.9	90	1990
United Kingdom	101	0.47	6,706	205	66.4	42	1997
Germany	96	0.54	5,944	234	61.9	44	1997
Canada	47	0.51	2,314	88	49.2	25	1999
Australia	36	0.58	2,106	55	58.5	20	2002
France	34	0.65	2,351	75	69.1	21	1993
Sweden	26	0.77	716	22	27.5	13	2008
New Zealand	25	0.52	1,425	32	57.0	18	1990
Spain	25	0.40	1,047	23	41.9	17	1992

Ps, number of publications; MCP, intercountry collaboration indices; GCS, global citation scores (times); GCS/Ps, average number of citations per paper; LCS, local citation score (times); SY, start year.

Among research institutions, the University of Chinese Academy of Sciences ranked the first with 156 documents; other active research institutions with higher production were the Institute of Botany (65 documents), Northwest A&F University (53 documents), University of Oklahoma (53 documents), Institute of Geographic Sciences and Natural Resources Research (52 documents), and Lawrence Berkeley National Laboratory (50 documents). It is noteworthy that Colorado State University achieved the highest GCS/Ps values of 141.2 (Table 3), primarily due to the large citation from two documents (Parton et al., 2007; Schimel et al., 2007), while the high GCS/Ps of 70.5 from the University of Oklahoma was attributed to the article “Phylogenetic molecular ecological network of soil microbial communities in response to elevated CO_2_” published on Mbio in 2011 (Zhou et al., 2011). In terms of influence level, the University of Chinese Academy of Sciences ranked the first with an *h*-index of 34, followed by the Institute of Botany with 31 and Colorado State University with 28 (Table 3). The analysis of active research institutions reveals that China emerges as a major contributor in the field of microbial-mediated grassland carbon cycling. This suggests that China holds a prominent position and contributes significantly to the advancements and research efforts in this field.

**Table 3:** The top 10 most productive institutions in the field of microbial-mediated grassland carbon cycling (Scopus, from 1990 to 2022).

**Institution**	**Country**	**Ps**	**GCS**	**LCS**	**GCS/Ps**	***h*-index**	**SY**
University of Chinese Academy of Sciences (UCAS)	China	156	4,336	221	27.8	34	2007
Institute of Botany (IOB)	China	65	3,653	231	56.2	31	2004
Northwest A&F University (NWAFU)	China	53	2,039	88	38.5	24	2005
University of Oklahoma (OU)	USA	53	3,739	215	70.5	27	2009
Institute of Geographic Sciences and Natural Resources Research (IGSNRR)	China	52	961	54	18.5	20	2007
Lawrence Berkeley National Laboratory (LBNL)	USA	50	2,548	164	51.0	25	2011
Institute of Applied Ecology (IAE)	China	44	2,389	96	54.3	23	2009
Tsinghua University (THU)	China	41	2,455	145	59.9	23	2011
Colorado State University (CSU)	USA	40	5,648	204	141.2	28	1991
Institute of Soil and Water Conservation (ISWC)	China	39	1,567	61	40.2	19	2009

Ps, number of publications; GCS, global citation scores (times); GCS/Ps, average number of citations per paper; LCS, local citation score (times); SY, start year.

##### 3.2.1.2 Collaboration network

**Figure 4:**
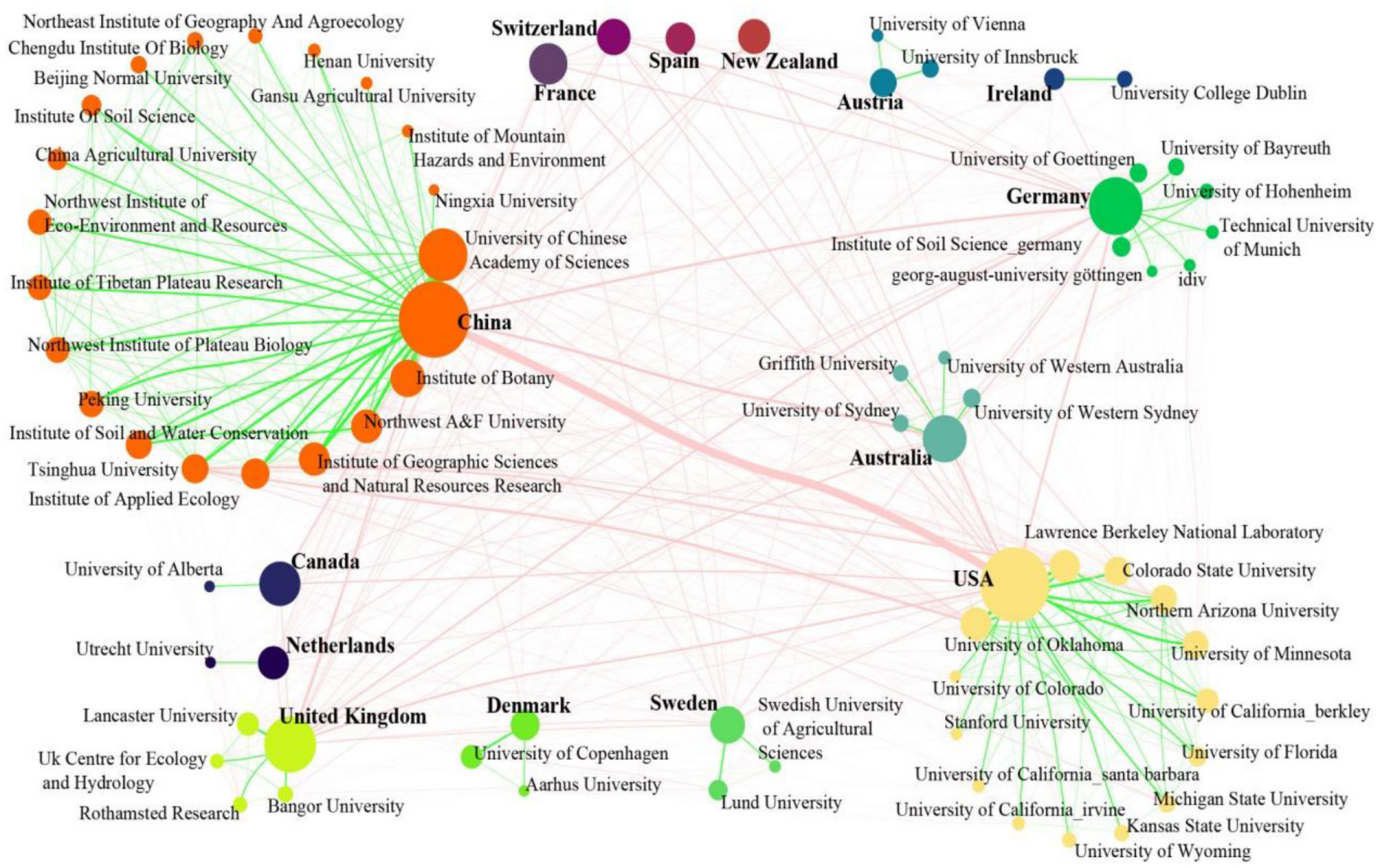
Co-occurrence network of countries and institutions in the field of microbial-mediated grassland carbon cycling (Scopus, from 1990 to 2022). The nodes of this network represent countries or institutions, and edges stand for their collaborative connection. The nodes are colored by countries, and size of node is determined by the number of documents produced by countries and institutions. The color of edge indicates whether the collaboration is national (blue) or international (red), and the width of edges is proportional to the number of collaborative documents.

To illustrate the academic collaboration in the field of microbial-mediated grassland carbon cycling between countries and institutions, a co-occurrence network was created. This network included institutions with more than 15 publications and their affiliated countries, along with the top 10 most productive countries in this field. The co-occurrence network was composed of 73 nodes (15 countries and 58 institutions) and 582 edges (97 edges among countries, 427 edges among institutions; Figure 4). China, the USA, the United Kingdom, and Germany were the significant academic contributors with the larger node. Those countries had extensive collaboration with almost all other countries, with the exception of Switzerland (co-operators: 11) and Ireland (10). The most significant collaboration relationships among countries were China–USA (133 documents), China–Australia (44 documents), China–Germany (40 documents), USA–Australia (34 documents), USA–Germany (32 documents), and USA–United Kingdom (30 documents). Most of those collaborations began in the first decade of the 21st century and reached their peak around the year 2020. Furthermore, China and the USA were the most influential countries in the co-occurrence network, while Australia, the United Kingdom, Sweden, Austria, Denmark, and Canada had rather low influence (Figure 4). The discrepancy may be caused by the low number of institutions from those countries participating in the collaboration network.

While the University of Minnesota may have been the first institution to study microbial-mediated grassland carbon cycling, the University of Chinese Academy of Sciences has emerged as the largest contributor in terms of publications in this field. Furthermore, the University of Chinese Academy of Sciences held the highest influence in the co-occurrence network, followed by the University of Oklahoma and Colorado State University. The University of Chinese Academy of Sciences closely collaborated with the Institute of Botany (39 documents), Northwest A&F University (19 documents), the Institute of Geographic Sciences and Natural Resources Research (34 documents), and the Institute of Applied Ecology (20 documents), among 19 national and 19 international collaborative institutions. Lawrence Berkeley National Laboratory and Tsinghua University were the most important national and international collaborators of the University of Oklahoma, respectively. The Colorado State University closely collaborated with the University of Minnesota, the University of Western Sydney, and the University of Wyoming.

##### 3.2.1.3 Research development in selected countries within given field

**Figure 5:**
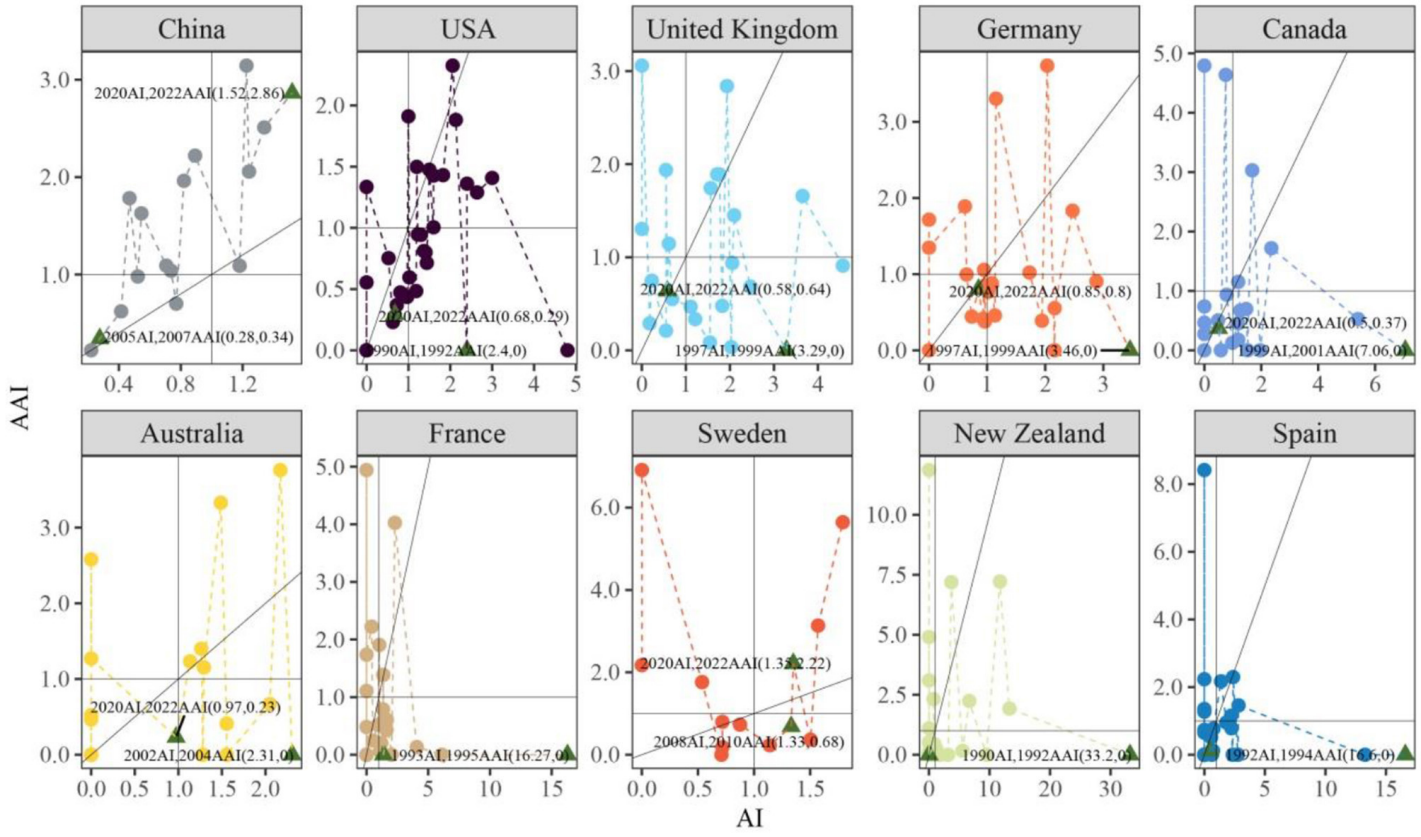
Line plots of AI and AAI for the top 10 most productive countries in the field of microbial-mediated grassland carbon cycling (Scopus, from 1990 to 2022). The plots were divided into four quadrants by horizontal line representing the AI index and vertical line representing the AAI index: points in the first quadrant present years where both AI and AAI indices are higher than global average level, points in the second quadrant present years where AI is lower than global average level but AAI is higher than global average level, points in the third quadrant present years where both AI and AAI indices are lower than global average levels, points in the fourth quadrant present years where AI is larger than global average level but AAI is lower than global average level. The first and last points of countries were marked with triangles and labeled with text.

The AI and AAI indexes were used to access the temporal change in research effort and academic importance for the top 10 most productive countries (Figure 5). Both the USA and New Zealand had earlier initial publication year and most points (1990, 31 points), followed by Spain (1992, 29 points) and France (1993, 28 points). On the contrary, China (2005, 16 points) and Sweden (2008, 13 points) had the fewest points due to their later initial publication year. Most points in the quadrant diagram of Canada (nine points, 40.9%), France (11 points, 39.3%), Spain (15 points, 51.7%), and New Zealand (13 points, 41.9%) were located in the third quadrant, which means that research effort and academic importance of these countries are lower than global average in most publication years, while most points from the USA (10 points, 32.3%), China (5 points, 31.2%), Australia (five points, 26.3%), and the United Kingdom (6 points, 25.0%) were positioned in the first quadrant, suggesting that their research effort and academic importance are larger than global average in most publication years (Figure 5). These points in the first quadrant of the USA and United Kingdom plots represented time scopes from 1994 to 2008 and 2000 to 2010, respectively. Conversely, points in the third quadrant corresponded to the time scope of 2014–2020 and 2013–2020. Interestingly, China and Australia exhibited opposite trend, with majority points in the first quadrant stemming from the scopes of 2013–2020 and 2011–2019. In contrast, points in the third quadrant for China and Australia were from earlier time scopes of 2005–2012 and 2003–2009 (Figure 5). These findings indicate that the research effort and academic impact of China and Australia in the field of microbial-mediated grassland carbon cycling have increased in recent years, and those of the USA and the United Kingdom have decreased in the last 10 years. In addition, when considering distance between points of a given country and the balance line, it could be observed that China and Australia were relatively close to the balance line, which indicates their research efforts are well balanced with their academic importance.

#### 3.2.2 Author analysis

A total of 6,380 authors participated in this study, with 47 of them publishing more than 10 documents. Zhou Jizhong emerged as the most productive author with 42 publications, followed by Kuzyakov Yakov (31 documents), Hobbie Sarah E. and Bardgett Richard D. (21 documents), and Luo Yiqi and Yang Yunfeng (20 documents; Table 4). The most productive authors represented nine research institutions, with five from the USA, two from Australia, and one each from Germany, the United Kingdom, China, and Austria (Table 4).

**Table 4:** Top 11 most productive authors in the field of microbial-mediated grassland carbon cycling (Scopus, from 1990 to 2022).

**Author**	**Institution**	**Country**	**Ps**	**GCS**	**LCS**	***h*-index**	**SY**
Zhou Jizhong	University of Oklahoma	USA	42	2,183	137	22	2011
Kuzyakov Yakov	University of Goettingen	Germany	22	1,169	49	15	2006
Hobbie Sarah E.	University of Minnesota	USA	21	2,451	136	18	1998
Bardgett Richard D.	University of Manchester	United Kingdom	21	2,239	105	17	1995
Luo Yiqi	Northern Arizona University	USA	20	1,875	106	16	2009
Yang Yunfeng	Tsinghua University	China	20	699	66	14	2013
Jones Davey L.	University of Western Australia	Australia	18	783	28	14	2007
Schuur Edward. A.G.	Northern Arizona University	USA	18	1,868	130	14	2009
He Zhili	University of Oklahoma	USA	18	1,772	108	17	2011
Richter Andreas	University of Vienna	Austria	17	901	54	12	2006
Dijkstra Feike A.	University of Sydney	Australia	17	787	45	12	2006

Ps, number of publications; GCS, global citation scores (times); LCS, lobal citation score (times); SY, start year.

**Figure 6:**
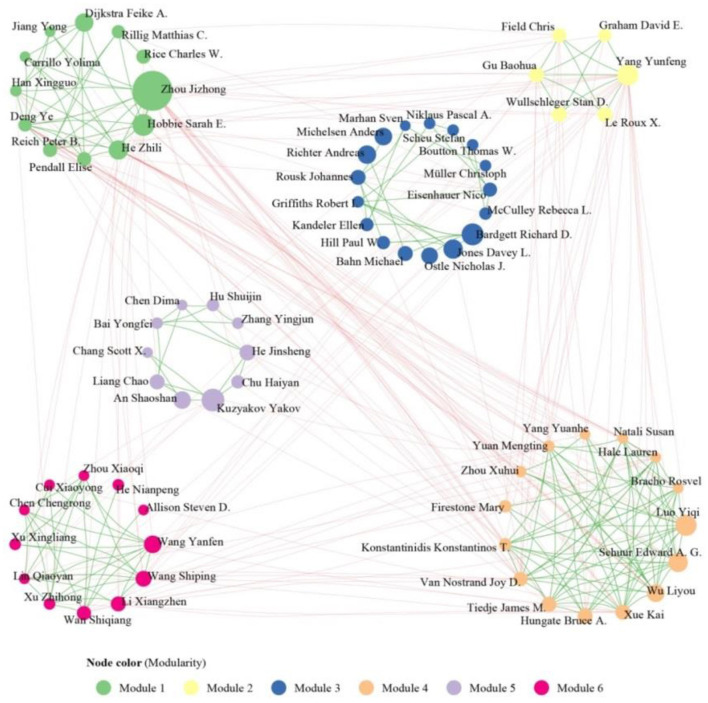
Collaboration network of authors (>8 documents) in the field of microbial-mediated grassland carbon cycling (Scopus, from 1990 to 2022). The nodes and edges stand for author and collaboration links between authors. The size of nodes is proportional to publications harbored by authors. Nodes were colored based on modularity.

Authors and their social relationships are crucial components of the research field. The interactive network formed by these authors and their social relationships plays a vital role in advancing knowledge and fostering research power. The cooperative relationship and teamwork effort among authors were explored by co-occurrence network of authors. These authors involved in this study established 31,150 cooperative relationships, indicating extension collaboration among researchers in the field of microbial-mediated grassland carbon cycling. To reduce the complexity and redundancy of collaboration network, Figure 6 depicted the cooperative relationship among authors who had more than eight documents and was composed of 72 authors and 333 collaborative links.

The co-occurrence network of authors was analyzed for modularity, resulting in organization of six distinct modules (Figure 6). Each module represented a group with substantial academic effect and noteworthy research achievement. Module 1 (colored in green) was led by Zhou Jizhong, Hobbie Sarah E., Dijkstra Feike A., and He Zhili. It consisted of 12 cooperators and 34 collaborative relationships. This team focuses on the impact of elevated CO_2_ and water availability on the composition, diversity, and function of the soil microbial community (Zhou et al., 2011; He et al., 2012; Ullah et al., 2021). Module 2, colored in light yellow and led by Yang Yunfeng, had the fewest authors (6), co-occurrence relationships (11), and academic products (11). This team focuses on the magnitude, rate, and stoichiometry of anaerobic organic matter decomposition from arctic tundra (Roy Chowdhury et al., 2015; Wang et al., 2019; Zheng et al., 2019). Module 3, colored by blue and led by Bardgett Richard D., Jones Davey L., and Richter Andreas, consisted of 17 authors and 25 collaboration relationships. It focused on exploring the relationship between microbial properties and the plant–soil interaction underlying grassland plant richness and productivity (Lange et al., 2015; Abrahão et al., 2022). The team of Luo Yiqi and Schuur Edward. A. G., highlighted in ochre color in collaboration network (module 4), had the biggest number of collaborations (71), indicating a strong and consistent collaborative relationship within the team. This team conducts an extensive study on the response of soil respiration and ecological respiration to permafrost thawing under warming conditions (Natali et al., 2014; Schädel et al., 2018; Schuur et al., 2021). They are responsible for the majority of academic results (57.14%) from 2012 to 2018. Module 5, led by Kuzyakov Yakov and composed of 10 authors and 17 collaborations, focuses on studying the contribution of microbial necromass and plant residuals to soil organic carbon level (Ma et al., 2018; Wang et al., 2021; Zhang et al., 2021; Yang et al., 2022). This team accounts for majority (66.7%) of collaborations in 2019–2022. Module 6, colored by magenta, consisted of 12 authors and 31 collaborations. It focuses on the methane oxidation and methanotrophic community in semi-arid grasslands (Ma et al., 2016; Kou et al., 2017).

### 3.3 Intellectual base

The co-citation analysis was used to uncover the intellectual foundation and identify the most impactful documents in the field of microbial-mediated grassland carbon cycling. The importance of documents was assessed using two indicators: local citation score (LCS) and betweenness centrality (BC). The former measures the level of attention that the document has received and reflects its contribution to certain fields. The latter was defined as the ratio of number of the shortest path going through given node to the sum of shortest path in co-citation network. The document is considered to have played a fundamental and significant role in certain discipline if it exhibits high local citation score and betweenness centrality. In this study, it was found that 75 documents have a local citation score of more than 10, and 10 documents exhibit a higher value of betweenness centrality (>15) in the co-citation network. Notably, eight documents stood out by demonstrating both high LCS (from 10 to 64) and BC (from 23 to 96.3) and collectively formed intellectual foundation in the field of microbial-mediated grassland carbon cycling (Table 5).

**Table 5:** The important documents with high citation frequency and betweenness centrality in the field of microbial-mediated grassland carbon cycling (Scopus, from 1990 to 2022).

**Author**	**Journal**	**Title**	**BC**	**GCS**	**LCS**	**PY**
Victoria J. Allison	*Soil Science Society of America Journal*	Changes in soil microbial community structure in a tallgrass prairie chronosequence	41.5	202	12	2005
Joshua Schimel	*Ecology*	Microbial stress–response physiology and its implications for ecosystem function	96.3	1,577	64	2007
Helen Gordon	*Soil Biology and Biochemistry*	Drying and rewetting effects on soil microbial community composition and nutrient leaching	53.5	307	21	2008
Kate M. Buckeridge	*Applied Soil Ecology*	Deepened snow alters soil microbial nutrient limitations in arctic birch hummock tundra	61.0	141	13	2008
M. Lavoie	*Journal of Geophysical Research: Biogeosciences*	Effects of elevated nitrogen and temperature on carbon and nitrogen dynamics in Alaskan arctic and boreal soils	35.0	84	12	2011
Eduardo Medina-Roldán	*Agriculture, Ecosystems & Environment*	Grazing exclusion affects soil and plant communities but has no impact on soil carbon storage in an upland grassland	35.5	144	11	2012
Seeta A. Sistla	*Nature*	Long-term warming restructures Arctic tundra without changing net soil carbon storage	36.0	308	22	2013
Susan M. Natali	*Ecology*	Permafrost degradation stimulates carbon loss from experimentally warmed tundra	23.0	107	10	2014

BC, betweenness centrality; GCS, global citation scores (times); LCS, local citation score (times); PY, publication year.

Furthermore, the co-citation network analysis revealed the evolution pattern of theme within intellectual foundation. The seminal literatures that have contributed to understanding the theme evolution of knowledge network in this field can be traced back to the documents titled “Changes in soil microbial community structure in a tallgrass prairie chronosequence,” which was published in the *Soil Science Society of America Journal* by Victoria J. Allison in 2005 (Table 5). This document is a fine specimen that focuses on understanding relationship behind the change in soil total organic carbon (TOC) content resulting from the conversion of land management practices in the 2000s. The results showed the prairie, previously used in tillage-based agriculture, experienced a significant accumulation of TOC and an increase in abundance ratio of fungi and bacteria. It is hypothesized that elevated TOC level is attributed to protection mechanism associated with stable soil structure offered by fungi, other than solely improving community metabolic efficiency (Allison et al., 2005). The critical document with the highest LCS (64) provides valuable insight into the physiological response of microorganisms at the organism level to environmental stress and their immediate impact on carbon flow at the ecosystem level through review methodology (Schimel et al., 2007) (Table 5). Other pivotal documents place great emphasis on shift of microbial community and function groups, as well as the allocation and fate of grassland carbon in the face of environmental stress (drought and rewetting, freezing, and experimental warming) based on experimental studies from 2008 to 2014 (Table 5).

### 3.4 Research hotspots

#### 3.4.1 Clustering analysis of high-frequency keyword

Cluster analysis in bibliometric research takes the co-occurrence frequency of keywords as subject of analysis and uses statistical methods to simply comprehensive network relationship into a small number of groups. The hierarchical clustering algorithm merges with each keyword to form extensive hierarchy of clusters based on the distance between them and treats the keywords as being more related to those nearby than further away in the tree dendrogram.

**Figure 7:**
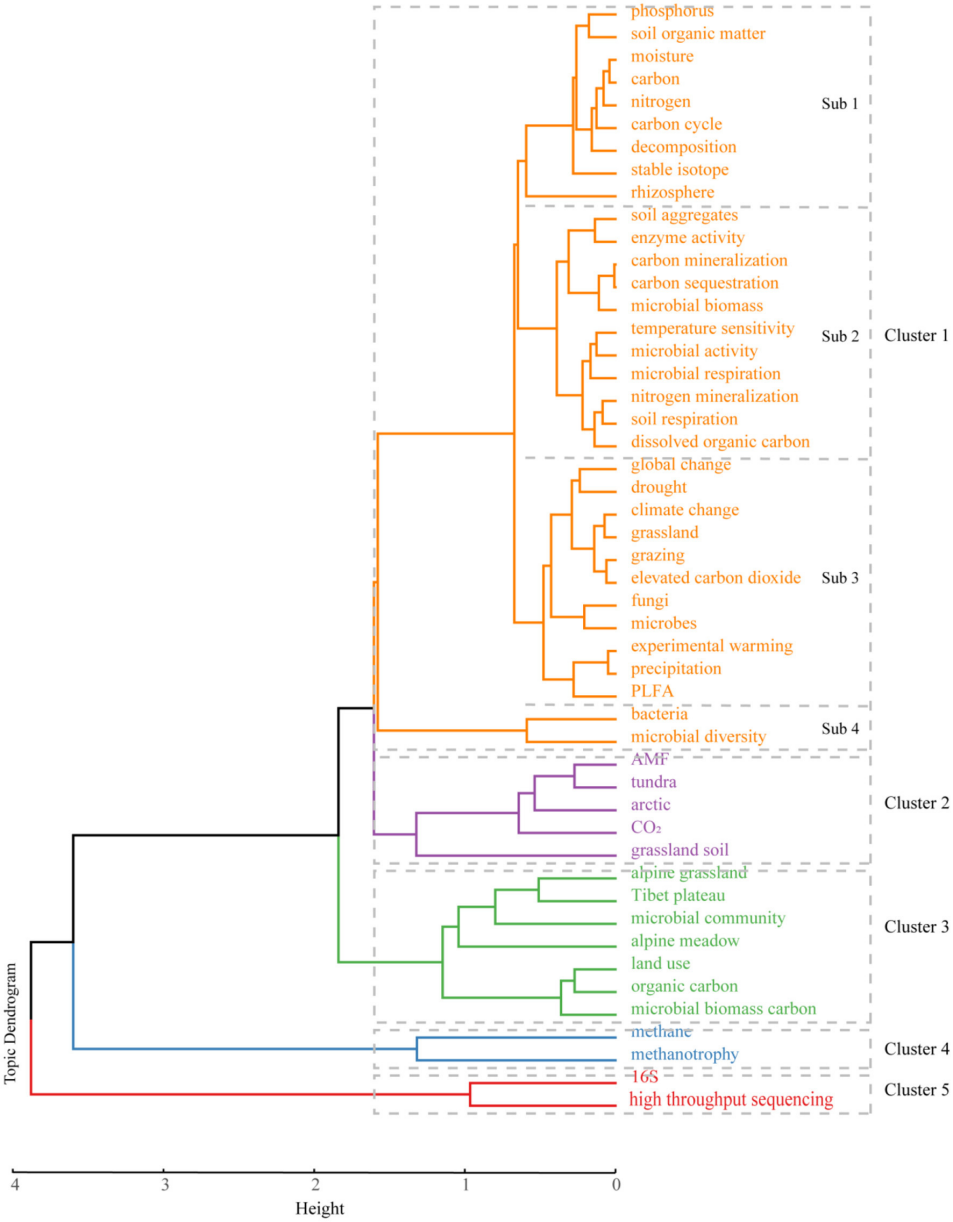
Tree dendrogram of hierarchical cluster analysis of high-frequency keywords in the field of microbial-mediated grassland carbon cycling (Scopus, from 1990 to 2022).

Those high-frequency keywords with an occurrence >20 were categorized into five clusters (Figure 7). Cluster 1 contained 33 high-frequency keywords, which were mainly divided into four subclusters. There were a total of 11 frequently occurring keywords within subclusters 1 and 4, including soil organic matter, decomposition, carbon cycle, bacteria, microbial diversity, and more. Moreover, subclusters 2 and 3 each included 11 high-frequency keywords. We noticed the high-frequency keywords involved in subcluster 3 mainly focus on grazing impacts, fungi, elevated carbon dioxide, experimental warming, climate change, drought, precipitation, and so on (Figure 7). In general, cluster 1 mainly focuses on the influence of microbial respiration on soil carbon storage and sequestration and the critical factors influencing microbial-driven carbon dynamics in grasslands (Allison et al., 2010; Sheik et al., 2011; Zhou et al., 2012; Nie et al., 2013; Guo et al., 2020). Warming typically amplifies soil microbial respiration and accelerates the depletion of soil carbon (Schmidt et al., 2011; Nie et al., 2013), despite a reduced temperature sensitivity in microbial respiration (Allison et al., 2010). The long-term warming (≥5 years) amplifies the ratio of ligninase to cellulase activity, resulting in the microbial breakdown of recalcitrant carbon compounds and a 14% decrease in the recalcitrant carbon pool in the topsoil (Chen et al., 2020). However, the elevation of atmospheric CO_2_ (eCO_2_) appears to have negligible impact on grassland soil carbon storge due to the constant abundance of the gene involved in the degradation of recalcitrant carbon (He et al., 2010; Trivedi et al., 2013). In addition, the precipitation regime plays a crucial role in the substantial accumulation and loss of carbon during drought and rewetting periods (Schimel et al., 2007). If precipitation increased, the enzyme activity, e.g., oxidase and nitrogen acquisition extracellular enzymes, are stimulated (Xiao et al., 2018). Precipitation also alters the composition and function of soil fungal communities and decreases the proportions of arbuscular mycorrhizal fungi but has no effect on the saprotroph (Huang et al., 2021). Furthermore, as an anthropogenic intervention in water resource supply, irrigation can have varying effects on microbial-driven carbon dynamics in grasslands depending on the climatic zone and duration of irrigation (Whitehead et al., 2018). Specifically, irrigation has the potential to reduce soil carbon stocks in arid zones (precipitation >575 mm) (Mudge et al., 2021), as well as in sites that have been irrigated for more than 12 years.

Previous study has established that these soil carbon stocks are sensitive to land management: grazing, species composition, and mineral nutrient availability can lead to losses or gains of soil carbon. As an important influencing factor, animal grazing suppresses microbial respiration by affecting belowground biomass and increasing soil compaction, particularly in areas with higher grazing intensity compared to natural grasslands (Li et al., 2024). The impact of grazing type and strength on carbon stock, sequestration, and flux among different pools of grassland soil often vary. For example, continuous grazing decreases plant height, plant species diversity, and live root biomass and increased individual density (Hoffmann et al., 2016). Summer grazing decreases the net carbon sink, but winter grazing increases it. Rotational grazing between the summer and winter seasons causes neutral climate feedback. Furthermore, under conditions of moderate grazing and warming, both summer and winter grazing have no significant effects on ecosystem CO_2_ exchange (Lv et al., 2020). In total, livestock grazing in grassland globally has been associated with an average decrease of 15% in soil organic carbon, although the extent of the decrease varies between 4.5% in the tropics and 22.4% in the temperature zone (Eze et al., 2018). The magnitude of carbon loss varies considerably across different grassland types, climatic conditions, and levels of grazing activity. Therefore, the impact of climate change and human activities on microbial respiration and carbon dynamics exhibits significant dependence on the specific context.

Cluster 2 was focused on the interaction between plants and microorganisms, including AMF, arctic, tundra, and other keywords (Figure 7). AMF are capable of establishing arbuscular mycorrhizal (AM) symbiosis with over 80% of terrestrial plant species (Smith and Read, 2010), providing their host with nutrients, alleviating plant abiotic stress, and increasing resistance to pathogens (Smith and Read, 2010; Jung et al., 2012). Consequently, those widely distributed organisms with well-established functions have garnered significant attention from researchers on their interactions with plants (Hoeksema et al., 2010; Grman, 2012; Neuenkamp et al., 2018). Additionally, AMF play a crucial role in transporting significant amounts of photosynthetic carbon to the soil. This carbon is either occluded within soil aggregates or stabilized in mineral-associated organic matter, thereby enhancing carbon sequestration (Kakouridis et al., 2024). The research findings have indicated variations in AMF among functionally distinct groups of plants. For instance, it is observed that root colonization and AMF hyphal abundance are higher in C4 plants compared to C3 plants (Hoeksema et al., 2010; Grman, 2012), and the C4 plant harbors a higher number of partners of AMF (Davison et al., 2020). Additionally, the strength of correlation between plants and AMF weakens during succession following the cessation of grassland management (Neuenkamp et al., 2018). In arctic habitats, characterized by nutrient-limited decomposition and recycling of nutrients slowed by low temperature, there is particular emphasis on interaction relationship between entire microbial community and plants. On the one hand, vegetation type has been found to strongly shape microbial count and community structure (Wallenstein et al., 2007), microbial respiration, and metabolic activity (Grogan and Jonasson, 2006). On the other hand, the psychrophiles and psychrotolerants of soil bacterial and fungal groups participate in the degradation of organic matter in a cold environment (Buckeridge and Grogan, 2008); the microbial biomass can also be rapid turnover on time scales of days to months in soil; and enhances nutrient availability to plants in the following growing season (Schmidt et al., 2007). The knowledge gained from these studies is highly informative for understanding the interaction between plants and microorganisms, as well as their role in microbial-mediated grassland carbon cycling. However, the study of carbon flux in the interactions between plants and microbes is often limited to qualitative research. Quantitative investigations are warranted to elucidate the fate and magnitude of carbon.

Cluster 3 dealt with organic carbon and included microbial biomass carbon, land use, Tibetan plateau, and other keywords (Figure 7). Microorganisms, as engineers of biogeochemical cycle on earth, assimilate parts of easily degraded or accessible molecular organic matter in soil for microbial biomass or otherwise mineralize it for gaining energy (Liang et al., 2017). Since microbial biomass has rapid responses to environmental change, it has been proposed as an indicator of environmental changes in soil and routinely measured and expressed as carbon contained in microbial biomass (microbial biomass carbon, C_mic_) (Anderson and Domsch, 1989; Broos et al., 2007). In this category, researchers frequently use the C_mic_ concentration or ratio of C_mic_ to soil organic carbon to explore ecological response to different land management regime (Wen et al., 2013; Hu et al., 2017). Their findings suggest that the value increases if the input of organic matter to the soil increases, and vice versa. After cell death and subsequent lysis and fragmentation of soil microbes, some cell residues (microbial necromass) are persistently present and comprise slow-cycling carbon in grassland soil combined with refractory component of plant litter. Similarly, land use practices, such as converting cropland to grassland and sowing legumes (Jia et al., 2022), applying organic and inorganic fertilizers (Li et al., 2020), and grazing exclusion (Qu et al., 2024), demonstrate their positive impact on facilitating microbial turnover, formation, and persistence of microbial necromass. Consequently, these grassland land management interventions contribute to increased soil carbon sequestration. Another research interest in this category is quantitative assessment of the contributions of microbial necromass to soil organic matter in grassland ecosystems. Meta-analysis of amino sugar data from a global dataset of 31 grassland soils reveals that microbial-derived organic carbon accounted for 62% of the total soil organic carbon (Liang et al., 2019). However, a separate synthesis analysis of 278 grassland soil samples presents a lower estimation, suggesting that 47% of the soil organic carbon originated from microbial necromass (Wang et al., 2021). Those results indicate that microbial necromass plays an important role in SOC accumulation and stabilization. To note, the accuracy of the estimation method depends on the conservation factor of bacterial-specific amino sugars, which is calculated using the carbon content of bacterial biomass and the ratio of gram-positive to gram-negative bacteria (Whalen et al., 2022). To improve accuracy, researchers should measure the gram-positive to gram-negative bacteria ratio in their samples and calculate a site-specific bacterial conversion factor. This approach enhances the estimation of microbial contributions to soil organic matter (SOM) and provides more reliable insights into microbial-mediated grassland carbon cycling.

In terms of methane-oxidizing bacteria and methane oxidation in grassland soil, only cluster 4 was involved (Figure 7). Due to numerous reports on the rapid CH_4_ uptake rate and methane oxidation potential through *in situ* observation and laboratory analysis (Bender and Conrad, 1992; Shrestha et al., 2012; Wei et al., 2015; Kou et al., 2017), researchers have been actively investigating the CH_4_ sink capacity of grassland. Regional estimates, using data from multiple literature resources, have reveal the CH_4_ consumption in grassland ecosystem in China is equivalent to that of forest, with high-frigid region of the Tibet plateau being the largest contributor (Wang et al., 2014). Furthermore, empirical models predict that grassland ecosystems contribute to annual CH_4_ consumption of ~4 Tg (3.73 ± 1.41, 4.40) (Yu et al., 2017; Xu et al., 2022), accounting for ~0.64% of global CH_4_ sink (Saunois et al., 2020). Those findings suggest grasslands can be considered as significant biotic sink for CH_4_. Furthermore, researchers have investigated the organisms inhabiting grassland soil that consumes atmospheric CH_4_, utilizing the kinetic characteristics of CH_4_ oxidation, labeling experiments, and phylogenetic marker analysis. Results show that aerobic methanotrophs can be divided into low- and high-affinity types (Bender and Conrad, 1992; Knief et al., 2003). The high-affinity type, particularly under low (2–10 ppmv) atmospheric CH_4_ concentrations, possesses a strong competitive advantage and is believed to response for atmospheric methane oxidation (Pratscher et al., 2018; Tveit et al., 2019). The high-affinity type, also known as upland soil cluster (USC), is further classified into USC Alphaproteobacteria (USCα) and Gammaproteobacteria (USCγ) (Kolb, 2009). The members of USCγ have been found in several soils with neutral to alkaline pH and are considered to be primary methanotrophs in alpine grassland soils (Knief, 2015; Deng et al., 2019). On the other hand, the members of USCα show preference for acidic grassland soil (Shrestha et al., 2012). Although the CH_4_ oxidization rate in grassland ecosystem is influenced by TOC and TN levels (Kou et al., 2017), the activity of CH_4_ oxidation is closely associated with gas diffusion. As a result, some researchers have proposed that grassland soil, when subjected to warming and drought conditions, may exhibit an increase in CH_4_ absorption. This is believed to occur due to a decrease in water content, which enhances gas diffusion in the soil (Rafalska et al., 2023). Understanding this phenomenon is important for comprehending the potential impacts of climate change on grassland ecosystems and their role in the global carbon cycle.

Cluster 5 investigated 16S rRNA and high-throughput sequencing (Figure 7). Due to the extremely large amount of niche resulting from high physical and chemical heterogeneity at small scale and microclimatic characteristics, grassland soil harbors complex and diverse microbial communities. Researchers in this cluster show considerable interest in the ecology and geography of microorganisms residing in grassland soil by means of culture-independent methods and uncover common and dominant bacterial phyla including Proteobacteria, Acidobacteria, Actinobacteria, Firmicutes, and Chloroflexi, as well as fungal taxa composed of Ascomycota, Basidiomycota, Glomeromycota, and Zygomycota in grassland soil at local (Barnard et al., 2013) and regional scales (Maestre et al., 2015; Prober et al., 2015; Chen et al., 2017). Furthermore, continuous developments in genomics technologies, such as high-throughput sequencing of 16S rRNA (Barnard et al., 2013; Prober et al., 2015), 18S rRNA (Dumbrell et al., 2011), 28S rRNA (Barnard et al., 2013), and internal transcribed spacer (ITS) region (Prober et al., 2015; Chen et al., 2017), and declining sequencing costs have generated the massive volume of dataset, linking changes in its high-resolution fingerprint of microbial community composition to an increasing variety of potential functions. However, due to the lack of sufficient fungal isolation and identification data support and the characteristics of high variability of the ITS region, valid identification of fungi remains a common bottleneck in the current practice (Wehner et al., 2014). Hence, obtaining more reference sequences of the ITS region through isolation and culturation of fungi is indeed a valuable approach to addressing this issue.

#### 3.4.2 Keyword bursts analysis

**Figure 8:**
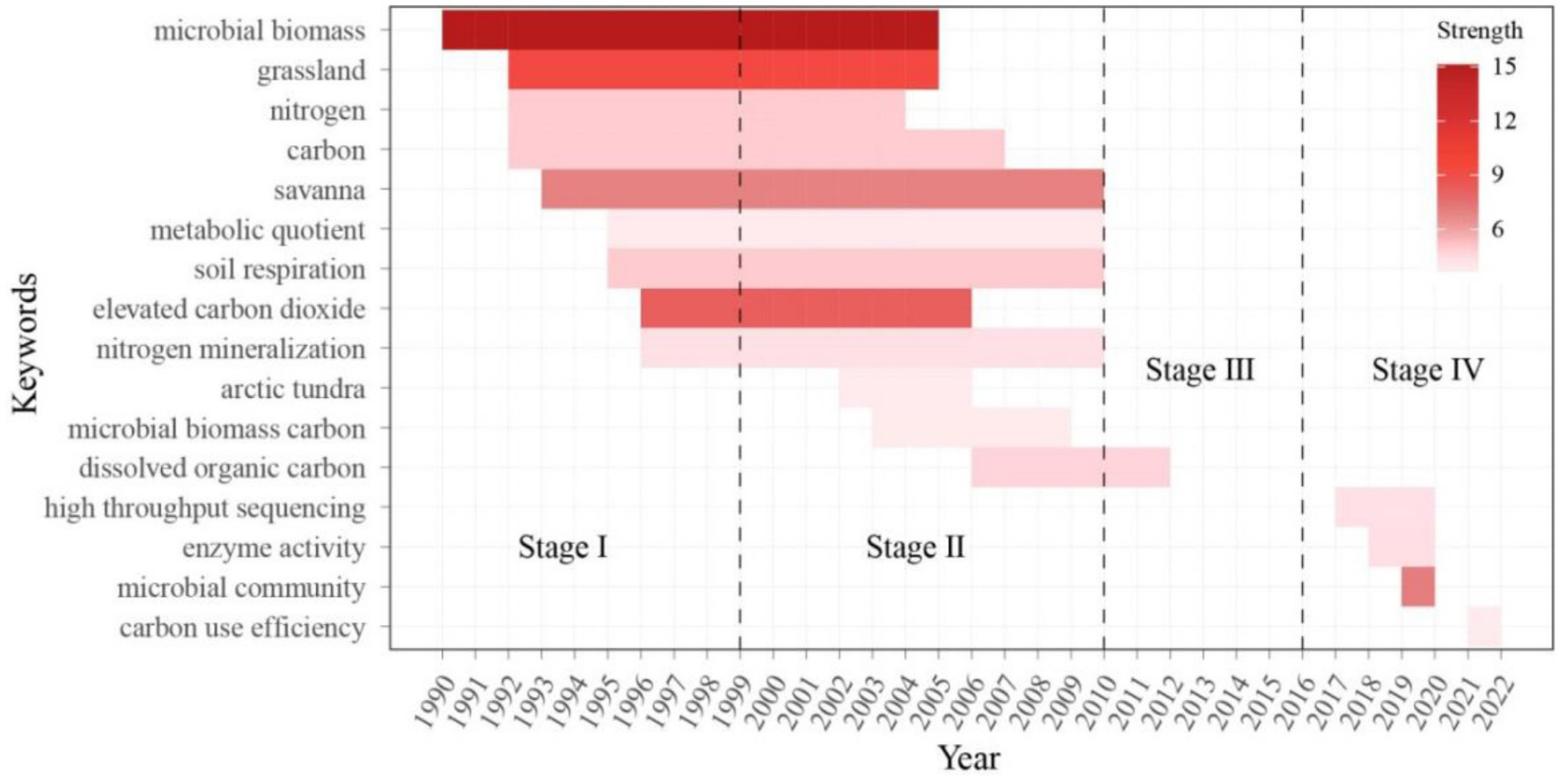
Bursting word analysis in the field of microbial-mediated grassland carbon cycling (Scopus, from 1990 to 2022). Each bursting word is visualized as a horizontal color bar with beginning and ending years, sorted by begging year. The color of horizontal bar is related to burst strength.

Keyword with burst means sudden increase in the frequency of word, suggesting that it receives much attention from the research community during a certain period. Therefore, burst detection is a useful tool to explore field frontiers and development advance. Figure 8 depicted the top 16 keywords with the strongest burst strength. Of them, nine keywords experienced burst from stage I to stage II, while only one keyword surpassed stage III. Notably, keywords including “carbon use efficiency,” “enzyme activity,” “microbial community,” and “high throughput sequencing” have shown the greatest increase since 2017 and represented the emerging active topics.

Extracellular enzymes play a crucial role in catalyzing the decomposition of polymeric organic matter into smaller, soluble molecules. This process results in the release of micro- and macronutrients into the environment, providing essential support for plants and microorganisms to growth. Therefore, the enzyme activity has been used as an important indicator to evaluate the effect of climate change (D'Alò et al., 2021) and land management (Dong et al., 2022) on ecological process and function under field experiments (Figure 8). Carbon use efficiency is another keyword that has experienced a burst in stage IV. It is related to soil organic carbon storage and carbon sequestration, which are affected by environmental condition, substrate availability, nutrient demand, and physical state and composition of microbial community (Ullah et al., 2021). Furthermore, we found high-throughput sequencing has produced an enormous volume of data and demonstrated a broad knowledge and understanding of microbial diversity, community structure, and ecological function. In fact, microbial community and related biogeochemical mechanisms have received increasing attention in earth system model projecting future climate and carbon cycling feedback, making it more closely match contemporary observation compared with tradition model without direct microbial control over soil carbon dynamics (Wieder et al., 2013) (Figure 8).

#### 3.4.3 Keyword evolution during four stages

**Figure 9:**
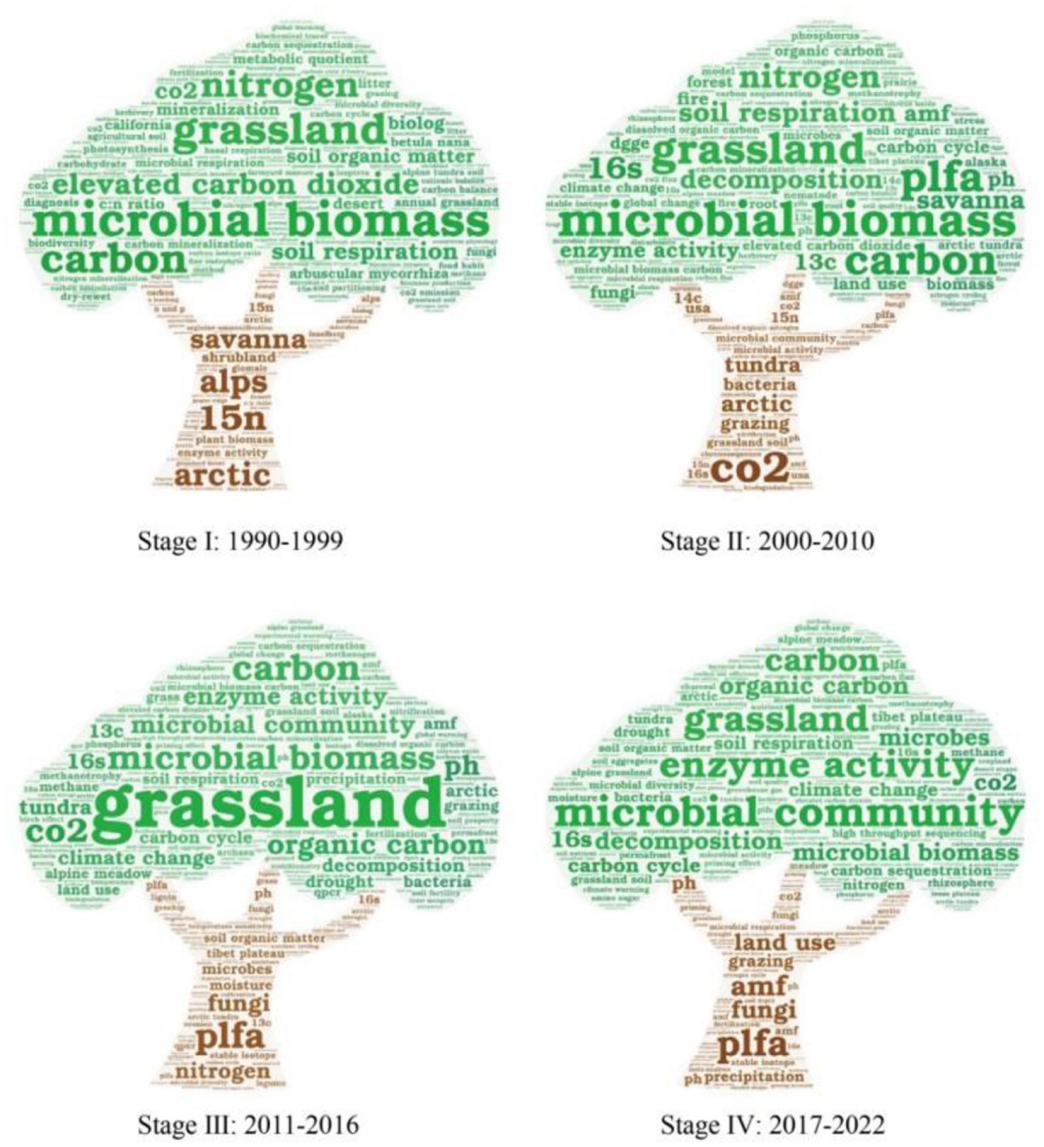
The evolution of “word cloud” in the field of microbial-mediated grassland carbon cycling (Scopus, from 1990 to 2022).

The high-frequency keyword evolution to four stages in the field of microbial-mediated grassland carbon cycling was presented in Figure 9. In the 1990s, the dominant research areas in the dataset were “microbial biomass” and “elevated CO_2_.” Various types of grassland, such as “savanna,” “annual grassland,” “shrubland,” and high-cold grassland from the “Alps” were also studied. At the beginning of this century, there was growing focus on carbon utilization in the process of carbon cycling. For example, “soil respiration” became the most-studied subject, followed by “microbial biomass” and “grassland.” “Decomposition” also played an important role in this stage. The “PLFA” method, an important tool for assessing the biological characteristics and metabolic status of soil microorganisms, significantly increased in popularity in 2008, following a consistent occurrence from 1999 to 2007. In stage III (2011–2016), research topics related to “enzyme activity” and “microbial community” started to emerge as hotspot topics. Additionally, research topics related to climate conditions, such as “climate change,” “moisture,” “drought,” “precipitation,” and “warming experiment,” began to gain prominence. In stage IV (2017–2022), the most popular research topics were “microbial community” and “enzyme activity.” Moreover, the frequency of topics associated with “high-throughput sequencing” experienced a notable surge during this period. The occurrence of such topics increased from 11 times in stage III (2011–2016) to 28 times in stage IV (2017–2022). The increased frequency of these topics suggests a wider adoption of high-throughput sequencing as a powerful tool for studying microbial diversity, community structure, and associated ecological functions. So far, attention is increasingly focusing on “alpine grassland,” “Tibet plateau,” and “land use.” Therefore, human activity and alpine grassland seem to be the primary subjects of microbial-mediated grassland carbon cycling as well as climate change.

#### 3.4.4 Keywords in different countries

**Figure 10:**
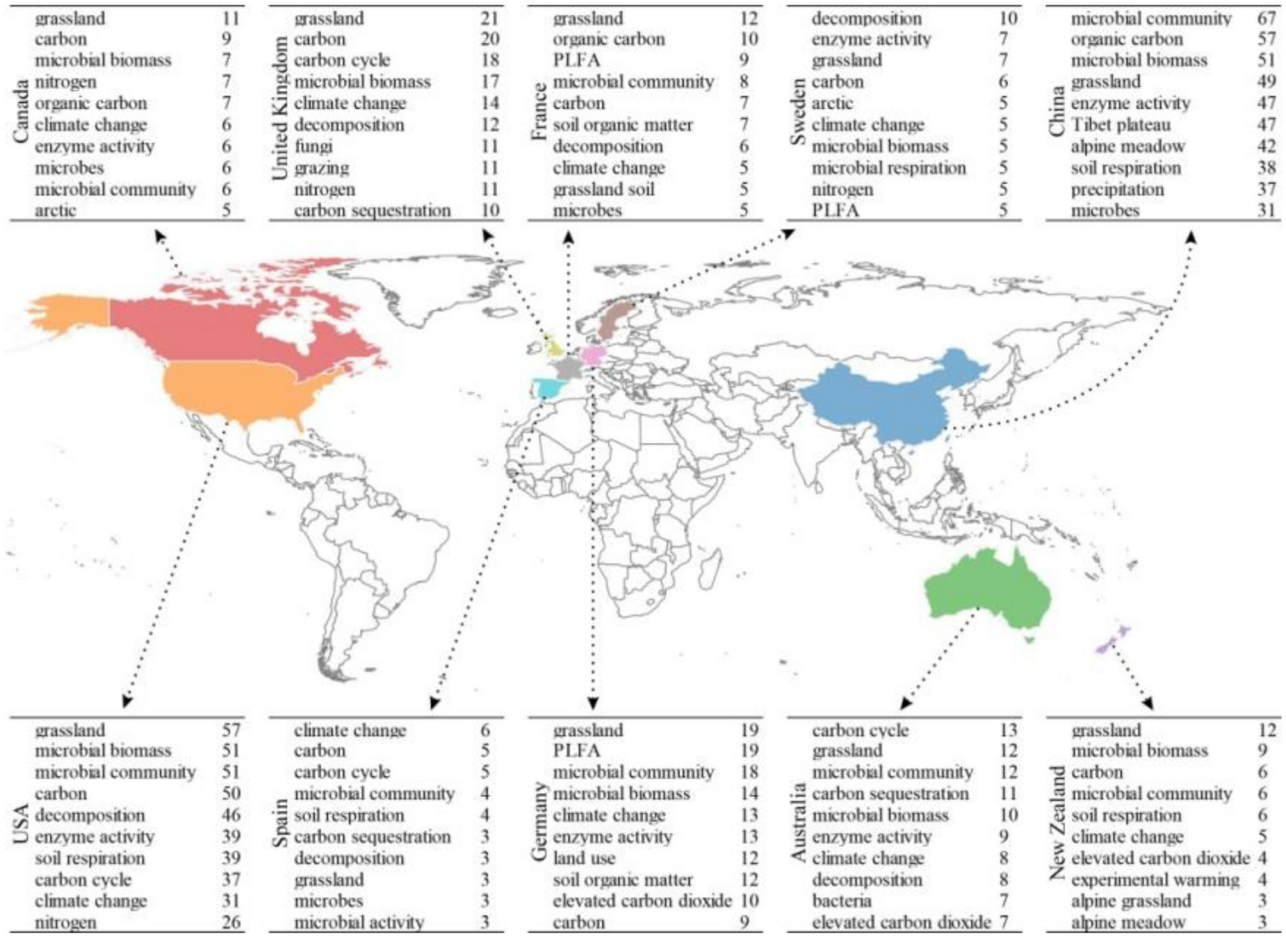
The 10 most frequently used keywords in the 10 most influential countries in the field of microbial-mediated grassland carbon cycling (Scopus, from 1990 to 2022). The table consists of three columns: country **(left)**, keyword **(middle)**, and frequency **(right)**.

Research on microbial-mediated grassland carbon cycling has garnered substantial attention worldwide due to its significance in the context of global climate change. However, due to variations in geographic features, climatic conditions, human history, and economic development, the subjects studied varied between regions. Figure 10 illustrated the 10 most influential countries by summarizing the 10 most frequently used keywords. It is evident that there were variations in the most frequently used keywords in different countries between 1990 and 2022. In China, the top keywords were “microbial community” (67 times), “organic carbon” (57 times), and “microbial biomass” (51 times). This indicates a strong emphasis on studying the composition and dynamics of microbial communities as well as the role of organic carbon and microbial biomass in grassland ecosystems in China. However, the most frequently used keywords in Sweden were “decomposition” (10 times) and “enzyme activity” (seven times), highlighting a focus on understanding the processes and mechanisms of organic matter breakdown and the involvement of enzymes in grassland decomposition. In Spain and Australia, “climate change” (six times) and “carbon cycle” (13 times) emerged as prominent keywords, indicating a research interest in examining the impacts of climate change on grassland ecosystems and the cycling of carbon within these systems. In general, these variations in frequently used keywords across different countries reflect the specific research priorities, environmental contexts, and challenges faced by each country in studying microbial-mediated grassland carbon cycling. It highlights the diversity of research interests and the importance of considering regional factors when studying ecological processes and responses to global environmental challenges.

## 4 Future directions

While microbial-mediated grassland carbon cycling studies have yielded various insights into how carbon cycling is related to microbial communities in grassland, there also remain important knowledge gaps, of which some of the most important ones are:

(1) A focus on long-term and comprehensive studies: in addition to the known influencing factors of the grassland ecosystem, the timescale over which changes in these factors are studied may also play a significant role. The relatively limited impact factors and short term of many field experiments conducted to date limit our ability to make realistic predictions about the response of microbial-mediated grassland carbon cycling to future changes. This is especially true when considering that short-term studies may yield misleading results compared to comprehensive studies that incorporate a wider range of factors and longer timescales.
(2) The response of microbial communities to elevated atmospheric carbon dioxide (eCO_2_) is a crucial component contributing to climate change feedback, yet it remains poorly understood. The influence of eCO_2_ on the structures and functions of soil microbial communities, as well as their response to carbon cycling in grasslands under comprehensive influencing factors, is a topic of ongoing research. Studies are being conducted to understand how eCO_2_ affects the composition, diversity, and metabolic activities of soil microbial communities, and how these changes impact carbon cycling processes in grassland ecosystems. By considering various influencing factors, researchers aim to gain a comprehensive understanding of the complex interactions between eCO_2_, soil microbial communities, and carbon dynamics in grasslands.
(3) The impact of climate change and human activities on microbial carbon flux remains highly uncertain. Factors such as carbon loss, CH_4_ intake, and carbon sequestration vary significantly across different grassland types, climatic conditions, and grazing intensity. Understanding and quantifying these complex interactions in various ecological contexts are essential for effective mitigation of increased atmospheric carbon.
(4) Current understanding of microbial carbon flux between different carbon pools in grassland ecosystems is primarily qualitative. Advancements can be made by utilizing new technologies and methodologies, such as isotope tracer and molecular fingerprinting approaches. Enhancing the quantitative method of amino sugar analysis by measuring the ratio of gram-positive to gram-negative bacteria and incorporating site-specific bacterial conversion factors will improve the accuracy of estimating microbial contributions to soil organic matter (SOM). Additionally, novel approaches combining amino sugar analysis with stoichiometric calculations, considering elemental ratios, can provide further insights into the microbial processes involved in carbon cycling.

Overall, addressing these knowledge gaps through further research and analysis will contribute to a more comprehensive understanding of microbial-mediated grassland carbon cycling and its implications for ecosystem functioning in the context of global climate change.

## 5 Conclusion

(1) Grassland ecosystems are some of the understudied ecosystems on Earth and contain a high diversity of microorganisms involved in microbial-mediated carbon cycling in the context of human activity and climate change. Many important questions remain to be explored in characterizing the given field through a bibliometric lens. To summarize, the intellectual landscape of this understudied field is thus a matter of the utmost importance. We provide the first quantitative global assessment on the field of microbial-mediated grassland carbon cycling by synthesizing 1,660 publications in the Scopus database (1999–2022) that discussed and unveiled an in-depth intellectual landscape and revealed significant trends, key contributors, noteworthy topics, and future directions.
(2) We found that the given domain was a multidisciplinary and interdisciplinary field that was mainly involved in the dominant subject categories of Agricultural and Biological Sciences, Environmental Science, and Immunology and Microbiology. The number of publications exhibited a consistent linear increase from stage I to stage IV within the given period of 1999 to 2022. The high-frequency keywords were clustered and mainly focused on the evolution of research hotspots within a given period.
(3) We unveiled seven core journals, eight documents, and six collaborative groups that serve as the cornerstone in the field of microbial-mediated grassland carbon cycling. Seven core journals can make significant contributions to a given field, and eight documents are the most important pieces of intellectual base. Six significant collaborative groups, consisting of high-yield authors, formed strong research networks and collaborations, resulting in impactful publications. The key contributors are China, the USA, Australia, and the United Kingdom, while active research institutions mainly come from China and the USA, such as the University of Chinese Academy of Sciences and the University of Oklahoma. China and the USA have the highest influence in the co-occurrence network.
(4) Keyword clustering involves five main research directions: (a) microbial respiration, (b) interaction relationships, (c) microbial biomass carbon, (d) methane oxidation, and (e) high-throughput sequencing. Meanwhile, carbon use efficiency, enzyme activity, microbial community dynamics, and high-throughput sequencing techniques have emerged as new research hotspots. These areas of investigation can provide valuable insights and guide future research on microbial-mediated grassland carbon cycling.
(5) In the context of global climate change, the existing literature's known conclusions may not provide a complete understanding of the carbon cycle process mediated by grassland microorganisms. Moving forward, it is crucial to comprehensively explore various potential factors in future studies, such as precipitation, warming, timescale, human activities, grazing intensity, management measures, land use patterns, and innovative technologies. These research areas open up opportunities for innovative approaches and methodologies to unravel the complexities of microbial-mediated carbon cycling and its implications for global climate change.

## Data availability statement

The original contributions presented in the study are included in the article/supplementary material, further inquiries can be directed to the corresponding authors.

## Author contributions

XX: Conceptualization, Data curation, Methodology, Software, Writing—original draft. TY: Conceptualization, Writing—review & editing. BM: Conceptualization, Funding acquisition, Writing—review & editing. DL: Formal analysis, Writing—review & editing. CL: Formal analysis, Writing—review & editing.
